# Investigation of the effect of TNF-α levels on infertility duration and infertility characteristics in endometriosis patients presenting with infertility complaints

**DOI:** 10.1186/s12905-026-04586-w

**Published:** 2026-06-01

**Authors:** Gülin Özuyar Şimşek, Özlem Uzunlar, Süleyman Özen, Tuba Çandar, Muzaffer Sancı, Ecem Dilan Duman

**Affiliations:** 1 Division of Gynecologic Oncology Surgery, İzmir City Hospital, İzmir, Türkiye; 2 Department of Obstetrics and Gynecology, Ankara Bilkent City Hospital, Ankara, Türkiye; 3 Department of Medical Biochemistry, Ufuk University, Ankara, Türkiye; 4 Department of Obstetrics and Gynecology, Private Ortadoğu Hospital, Ankara, Türkiye

**Keywords:** Endometriosis, Infertility, TNF-α, AMH, Inflammation, Ovarian reserve, Biomarker

## Abstract

**Background:**

Endometriosis is a chronic inflammatory disorder frequently associated with infertility. Tumor necrosis factor-alpha (TNF-α) is involved in inflammatory pathways; however, the clinical relevance of serum TNF-α in relation to infertility duration and ovarian reserve remains uncertain.

**Methods:**

This prospective comparative study included 70 infertile women aged 20–37 years: 35 women with clinically and/or radiologically diagnosed endometriosis and 35 age-matched infertile controls with unexplained infertility and no clinical or imaging evidence of endometriosis. Serum TNF-α was measured using enzyme-linked immunosorbent assay. Hormonal parameters, including anti-Müllerian hormone (AMH), were obtained from third-day menstrual-cycle samples retrieved from clinical records. Associations between TNF-α, infertility duration, AMH, and clinical characteristics were evaluated. Exploratory adjusted analyses were performed for log-transformed TNF-α and AMH.

**Results:**

Serum TNF-α levels were higher in the endometriosis group than in controls (median [IQR], 235.49 [124.33-390.31] vs. 64.58 [58.91–76.52] ng/L; *p* < 0.001). AMH levels were lower in the endometriosis group in unadjusted analysis (2.05 [1.67–3.41] vs. 3.30 [2.26–3.66] ng/mL; *p* = 0.029). After adjustment for age, BMI, smoking status, and previous surgical history, endometriosis group status remained associated with log-transformed TNF-α (β = 1.386, 95% CI 1.114 to 1.657; *p* < 0.001), whereas the association with AMH was attenuated (β=-0.824, 95% CI -1.817 to 0.169; *p* = 0.102). TNF-α was not associated with infertility duration or AMH within the endometriosis group.

**Conclusions:**

Serum TNF-α was elevated in infertile women with clinically and/or radiologically diagnosed endometriosis but was not associated with infertility duration, AMH, or selected clinical characteristics. The high discriminatory performance observed in ROC analysis should be interpreted as exploratory because of the modest sample size, clinical/radiologic case definition, potential selection bias, and lack of external validation. Serum TNF-α may reflect systemic inflammatory activity rather than infertility severity or ovarian reserve impairment.

## Introduction

Endometriosis is a chronic, systemic, and inflammatory disorder that affects approximately 10% of women of reproductive age and represents one of the leading causes of infertility [1–3]. Although the underlying mechanisms are not fully elucidated, immune dysregulation and a persistent inflammatory milieu are believed to contribute to disease pathogenesis and reproductive impairment [4–6]. Altered cytokine profiles have been implicated in disturbed folliculogenesis, impaired oocyte quality, and defective endometrial receptivity, all of which may reduce fertility potential in affected women [4–6].

Among pro-inflammatory mediators, tumor necrosis factor-alpha (TNF-α) has attracted considerable attention because of its involvement in ectopic endometrial implantation, angiogenesis, and immune activation [6–9]. Several studies have reported elevated TNF-α concentrations in serum and/or peritoneal fluid of women with endometriosis, supporting its role as a marker of inflammatory activation [7–9]. However, the clinical relevance of serum TNF-α remains controversial, and available studies have produced inconsistent findings regarding its association with infertility severity, reproductive outcomes, and ovarian reserve [8–10].

A major limitation of the current literature is that many biomarker studies have compared women with endometriosis with healthy fertile controls, rather than focusing specifically on infertile populations. This design may not clarify whether a biomarker is related to infertility-related clinical characteristics or simply reflects the presence of endometriosis. In addition, systemic cytokine levels may not accurately represent the peritoneal or follicular microenvironment, which is more directly involved in reproductive processes [10, 11].

The relationship between inflammatory activity and ovarian reserve is also incompletely defined. Anti-Müllerian hormone (AMH) is widely used as a marker of ovarian reserve, but ovarian reserve impairment in endometriosis may be multifactorial and influenced by disease-related structural damage, previous ovarian surgery, and local inflammatory changes. Therefore, the combined assessment of serum TNF-α and AMH in infertile women with endometriosis may help clarify whether systemic inflammatory activity is associated with ovarian reserve parameters.

Accordingly, this study aimed to compare serum TNF-α and AMH levels between infertile women with clinically and/or radiologically diagnosed endometriosis and age-matched infertile controls without evidence of endometriosis, and to evaluate whether serum TNF-α was associated with infertility duration, AMH, and selected clinical characteristics. Given the observational cross-sectional design, the study was intended to assess associations rather than causal effects.

## Materials and methods

### Study design and setting

This prospective comparative observational study was conducted at the infertility outpatient clinic of Ankara Bilkent City Hospital, Türkiye. The study protocol was approved by the Ankara City Hospital 2nd Clinical Research Ethics Committee (approval no. E2-22-2275; August 17, 2022). Written informed consent was obtained from all participants before enrollment. The manuscript was prepared in accordance with relevant reporting principles for observational studies.

### Study population and diagnostic criteria

The study population consisted of infertile women aged 20–37 years who presented to the infertility outpatient clinic. A total of 70 women were included in the final analysis, comprising 35 women in the endometriosis group and 35 women in the control group.

The endometriosis group consisted of infertile women followed with a diagnosis of endometriosis based on clinical and/or radiological findings and who had failed to conceive despite at least 6 months of unprotected intercourse. The 6-month criterion was used because women with suspected or established endometriosis are commonly evaluated earlier in infertility clinics due to the known association between endometriosis and impaired fertility. This definition may reduce comparability with studies using a conventional 12-month definition of infertility.

Endometriosis was diagnosed clinically and/or radiologically according to routine institutional practice and the structured assessment used in the endometriosis and chronic pelvic pain outpatient clinic. Clinical assessment included symptoms suggestive of endometriosis, such as cyclic pelvic pain, dysmenorrhea, periovulatory pain, chronic non-cyclic pelvic pain, dyspareunia, dyschezia, and dysuria, after exclusion of alternative causes. Pelvic examination findings suggestive of endometriosis included tenderness during bimanual palpation of the anterior cul-de-sac, pouch of Douglas, or adnexa, fixed uterus, and painful uterine mobility. Radiological assessment was based primarily on transvaginal and/or abdominal ultrasonography and pelvic magnetic resonance imaging when clinically indicated.

Ultrasonographic evaluation for endometriosis and endometrioma was performed using a 5-MHz transvaginal transducer with a Voluson S10 ultrasound system (General Electric, Germany, 2018). Surgical and histopathological confirmation was not available for the study cohort. Therefore, the endometriosis group should be interpreted as a clinically and/or radiologically diagnosed cohort rather than a surgically confirmed or histologically verified endometriosis cohort.

The control group consisted of age-matched infertile volunteers aged 20–37 years who presented to the same infertility outpatient clinic with infertility complaints during the study period. These women had unexplained infertility, no clinical or radiological diagnosis of endometriosis, and no anatomical pelvic pathology detected during routine gynecological evaluation. The control group was not composed of healthy fertile women, but of infertile women without evidence of endometriosis according to clinical and imaging assessment.

Women were excluded if they refused to participate, were outside the predefined age range, had male-factor infertility, had any known systemic comorbidity, active infectious or inflammatory disease, autoimmune disease, malignancy, or additional gynecological or pelvic pathology that could confound the interpretation of inflammatory markers or infertility-related parameters.

### Clinical evaluation

All participants underwent detailed gynecological evaluation. Demographic and reproductive variables including age, body mass index (BMI), gravidity, parity, abortion history, smoking status, infertility duration, previous surgery, and hysterosalpingography findings were recorded. In women with endometriosis, available clinical and imaging-based features including endometrioma presence, laterality, recurrence, and deep infiltrating endometriosis were evaluated. Formal surgical staging systems such as rASRM or ENZIAN were not available because surgical staging was not performed for all participants.

### Blood sampling and TNF-α measurement

Peripheral venous blood samples of approximately 10 mL were obtained from all participants. Blood samples were collected into yellow-capped vacuum gel tubes and transported to the laboratory within 30 min. Samples were centrifuged at 3600 rpm for 10 min using a Nüve NF800 centrifuge. The serum fraction was separated, transferred into Eppendorf tubes, and stored at -80 °C until analysis.

Serum TNF-α concentrations were measured at the Ufuk University Biochemistry Laboratory using a commercially available Human Tumor Necrosis Factor alpha ELISA kit manufactured by Bioassay Technology Laboratory (China; catalog no. E0082Hu). Optical measurements were obtained using a HEALES MB-530 microplate reader. According to the manufacturer’s specifications, the intra-assay and inter-assay coefficients of variation were < 8% and < 10%, respectively, the minimum detectable concentration was 1.52 ng/L, and the assay range was 3-900 ng/L. Results were reported in ng/L.

### Hormonal and other laboratory parameters

Hormonal parameters, including FSH, LH, estradiol (E2), and AMH, were retrieved from the hospital information management system. FSH, LH, estradiol, and AMH values were obtained from blood samples collected on the third day of the menstrual cycle as part of routine infertility evaluation. All routine laboratory measurements were performed at the Central Laboratories of Ankara City Hospital.

### Sample size calculation

Sample size estimation was performed using data derived from a previous study by Othman et al. [12]. Based on the reported between-group difference, the effect size was calculated as 0.79. Assuming a two-sided type I error of 0.05 and a statistical power of 90%, the minimum required sample size was calculated as 35 participants per group using R software version 3.6.1.

### Statistical analysis

Statistical analyses were performed using SPSS version 22.0 (IBM Corp., Armonk, NY, USA). Continuous variables were assessed for distribution using the Kolmogorov-Smirnov test and visual inspection. Variables with non-normal distribution are presented as median and interquartile range (IQR), while categorical variables are expressed as number and percentage. Between-group comparisons of continuous variables were performed using the Mann-Whitney U test. Categorical variables were compared using Pearson’s chi-square test or Fisher’s exact test, as appropriate. Correlations were evaluated using Spearman’s correlation analysis.

Receiver operating characteristic (ROC) curve analysis was used to explore the discriminatory performance of selected markers. The ROC analysis was considered exploratory because of the modest sample size, clinical/radiological case definition, and absence of external validation.

Because previous surgical history and smoking status differed significantly between groups, exploratory adjusted linear regression analyses were performed. Log-transformed TNF-α was used as the dependent variable because of its skewed distribution. Separate models were constructed for log-transformed TNF-α and AMH, including group status, age, BMI, smoking status, and previous surgical history as covariates. Given the sample size, these adjusted analyses were interpreted cautiously. A p value of < 0.05 was considered statistically significant. No formal adjustment was made for multiple comparisons; therefore, secondary analyses should be interpreted as exploratory.

## Results

A total of 70 infertile women were included in the final analysis, comprising 35 women with clinically and/or radiologically diagnosed endometriosis and 35 age-matched infertile controls without clinical or imaging evidence of endometriosis. Baseline demographic and clinical characteristics are shown in Table 1. Age, BMI, gravidity, parity, abortion history, and infertility duration were not significantly different between groups. Previous surgery was more frequent in the endometriosis group than in controls (18/35 [51.4%] vs. 7/35 [20.0%], *p* = 0.012), whereas smoking was less frequent in the endometriosis group (7/35 [20.0%] vs. 17/35 [48.6%], *p* = 0.022).

**Table 1:** Baseline demographic and clinical characteristics of the study population

Variable	Endometriosis group (*n* = 35)	Control group (*n* = 35)	*P* value
Age, years	30.00 [28.00–34.00]	28.00 [26.00–32.00]	0.120
BMI, kg/m²	23.00 [20.95–25.25]	24.20 [22.20–26.60]	0.145
Gravidity	0.00 [0.00–1.00]	0.00 [0.00-0.50]	0.295
Parity	0.00 [0.00–0.00]	0.00 [0.00–0.00]	0.161
Abortion	0.00 [0.00-0.50]	0.00 [0.00–0.00]	0.844
Infertility duration, months	30.00 [12.00–48.00]	24.00 [18.00–42.00]	0.830
Previous surgery, n (%)	18 (51.4)	7 (20.0)	0.012
Smoking, n (%)	7 (20.0)	17 (48.6)	0.022
Normal HSG, n (%)	30 (85.7)	35 (100.0)	0.470

Continuous variables are presented as median [interquartile range]. Categorical variables are presented as n (%)

Clinical and laboratory characteristics are shown in Table 2. Within the endometriosis group, endometrioma was present in 34 women (97.1%), deep infiltrating endometriosis in 7 women (20.0%), and recurrence in 7 women (20.0%). Disease lateralization was unilateral in 22 women (62.9%) and bilateral in 13 women (37.1%). FSH, LH, and estradiol levels did not differ significantly between groups. AMH was lower in the endometriosis group than in controls in the unadjusted analysis (median [IQR], 2.05 [1.67–3.41] vs. 3.30 [2.26–3.66] ng/mL; *p* = 0.029). Serum TNF-α levels were higher in the endometriosis group than in controls (235.49 [124.33-390.31] vs. 64.58 [58.91–76.52] ng/L; *p* < 0.001).

**Table 2:** Disease phenotype and laboratory findings

Variable	Endometriosis group (*n* = 35)	Control group (*n* = 35)	*P* value
Endometrioma present, n (%)	34 (97.1)	-	-
Bilateral disease, n (%)	13 (37.1)	-	-
Recurrence, n (%)	7 (20.0)	-	-
DIE, n (%)	7 (20.0)	-	-
FSH	6.90 [5.33–9.25]	7.20 [5.85-8.00]	0.991
LH	5.30 [3.95–5.95]	4.80 [3.70–7.55]	0.769
E2	57.00 [31.00–71.00]	44.00 [28.50–63.50]	0.224
AMH, ng/mL	2.05 [1.67–3.41]	3.30 [2.25–3.66]	0.029
TNF-α, ng/L	235.49 [124.33-390.31]	64.58 [58.91–76.52]	< 0.001

Laboratory variables are presented as median [interquartile range]. *DIE* Deep infiltrating endometriosis

Exploratory ROC analysis is summarized in Table 3. TNF-α showed high discriminatory performance in this sample, with an AUC of 0.979. Using a cut-off value of > 88.02 ng/L yielded 97.1% sensitivity and 97.1% specificity. Because of the modest sample size, single-center recruitment, clinical/radiological case definition, and lack of external validation, these findings were interpreted as exploratory rather than evidence of clinical diagnostic utility.

**Table 3:** Exploratory diagnostic performance of selected markers for endometriosis group

Marker	Cut-off	AUC	95% CI	Sensitivity (%)	Specificity (%)	*P* value
FSH	> 8.3	0.503	0.381–0.625	31.4	85.7	0.968
LH	≤ 6.5	0.515	0.392–0.636	80.0	45.7	0.838
E2	> 51	0.593	0.469–0.709	51.4	68.6	0.174
AMH	≤ 2.05	0.652	0.530–0.763	51.4	82.9	0.029
TNF-α	> 88.02	0.979	0.912–0.998	97.1	97.1	< 0.001
Infertility duration, months	≤ 12	0.504	0.382–0.626	31.4	77.1	0.949

ROC results are exploratory and were not externally validated. *AUC* Area under the curve, *CI* Confidence interval

Exploratory adjusted analyses are shown in Table 4. After adjustment for age, BMI, smoking status, and previous surgical history, endometriosis group status remained associated with log-transformed TNF-α (β = 1.386, 95% CI 1.114 to 1.657; *p* < 0.001). In contrast, the association between endometriosis group status and AMH was attenuated and was no longer statistically significant after adjustment (β=-0.824 ng/mL, 95% CI -1.817 to 0.169; *p* = 0.102).

**Table 4:** Exploratory adjusted analyses for TNF-α and AMH

Dependent variable	Main predictor	β coefficient	95% CI	*P* value
Log-transformed TNF-α	Endometriosis group	1.386	1.114 to 1.657	< 0.001
AMH	Endometriosis group	-0.824	-1.817 to 0.169	0.102

Models were adjusted for age, BMI, smoking status, and previous surgical history. TNF-α was log-transformed because of skewed distribution

Association analyses within the endometriosis group are summarized in Table 5. Serum TNF-α was not significantly correlated with infertility duration, AMH, FSH, LH, or estradiol. In subgroup analyses, TNF-α levels did not differ significantly according to disease laterality, recurrence, presence of deep infiltrating endometriosis, or infertility-duration subgroup.

**Table 5:** TNF-α association analyses within the endometriosis group

Analysis	Result	*P* value
Correlation with infertility duration	*r* = -0.133	0.448
Correlation with AMH	*r* = -0.058	0.739
Correlation with FSH	*r* = -0.202	0.245
Correlation with LH	*r* = -0.147	0.398
Correlation with E2	*r* = -0.108	0.535
Unilateral vs. bilateral disease	170.66 [116.26-305.48] vs. 347.78 [177.92-489.77]	0.091
Recurrence yes vs. no	123.52 [109.52-295.66] vs. 245.93 [138.02-407.84]	0.300
DIE yes vs. no	249.14 [109.52-485.56] vs. 221.82 [126.73-386.64]	0.952
Infertility duration 12–35 vs. ≥ 36 months	313.76 [146.24-441.01] vs. 173.99 [112.70-276.34]	0.176

Correlation analyses used Spearman’s correlation. Subgroup values are presented as median [interquartile range]

## Discussion

In this prospective comparative study of infertile women, serum TNF-α levels were higher in women with clinically and/or radiologically diagnosed endometriosis than in infertile controls, whereas AMH levels were lower in the endometriosis group in unadjusted analysis. However, serum TNF-α was not associated with infertility duration, AMH, or selected clinical features within the endometriosis group. After adjustment for age, BMI, smoking status, and previous surgical history, the association between endometriosis group status and log-transformed TNF-α persisted, whereas the AMH difference was attenuated. These findings suggest that serum TNF-α may reflect systemic inflammatory activity in clinically apparent endometriosis but does not appear to function as a marker of infertility duration or ovarian reserve impairment in this cohort.

Endometriosis is increasingly recognized as a chronic inflammatory disease characterized by immune dysregulation and altered cytokine signaling [1–6]. The elevated serum TNF-α levels observed in the present study are consistent with prior reports suggesting inflammatory activation in women with endometriosis [7–9]. Nevertheless, the lack of correlation between serum TNF-α and infertility duration or AMH suggests that systemic TNF-α alone may not adequately capture reproductive burden or ovarian reserve status.

A key interpretation of these findings relates to the compartmentalized nature of inflammation in endometriosis. Serum cytokine concentrations may differ substantially from local cytokine activity within the peritoneal cavity, follicular fluid, or ovarian microenvironment. These local compartments may be more directly involved in folliculogenesis, oocyte quality, fertilization, and implantation [13, 14]. Therefore, the absence of a serum TNF-α-AMH correlation should not be interpreted as evidence that inflammatory pathways are unrelated to ovarian reserve. Rather, it indicates that a single systemic measurement may be insufficient to represent the local inflammatory processes most relevant to fertility outcomes.

The unadjusted reduction in AMH among women with endometriosis should also be interpreted cautiously. Previous surgery was more frequent in the endometriosis group, and ovarian surgery is a recognized factor that may affect ovarian reserve. After adjustment for age, BMI, smoking status, and previous surgical history, the association between group status and AMH was attenuated and no longer statistically significant. This suggests that ovarian reserve findings in this cohort may not be attributable to endometriosis alone and may partly reflect surgical burden or other unmeasured confounders. Consequently, our results do not support a direct relationship between serum TNF-α and ovarian reserve depletion.

The ROC analysis demonstrated a very high AUC for serum TNF-α in discriminating women with endometriosis from infertile controls in this sample. However, this result must be interpreted with considerable caution. A single systemic cytokine would not typically be expected to show such high diagnostic performance in a chronic inflammatory disorder, especially in a small cohort without external validation. Possible explanations include selection of clinically apparent cases, limited representation of minimal or asymptomatic disease, single-center recruitment, biological variability, and assay-related factors. Therefore, the ROC findings should be regarded as exploratory and hypothesis-generating, not as evidence that TNF-α is ready for use as a standalone diagnostic biomarker.

The observational cross-sectional design also precludes causal interpretation. Elevated TNF-α should not be interpreted as causing infertility, prolonged infertility duration, or ovarian reserve decline. The appropriate interpretation is that serum TNF-α was higher in the endometriosis group but was not proportionally associated with infertility-related clinical or hormonal parameters within that group.

Several limitations should be acknowledged. First, endometriosis was not surgically or histopathologically confirmed in the study cohort. Diagnosis was based on clinical evaluation and imaging findings obtained during routine infertility and endometriosis clinic assessment. This reflects real-world clinical practice but introduces a risk of disease misclassification, particularly for minimal, superficial, early-stage, or asymptomatic endometriosis. Similarly, the absence of surgical staging prevented the use of standardized classification systems such as rASRM or ENZIAN, limiting stage-specific analyses. Second, the control group consisted of infertile women with unexplained infertility rather than healthy fertile controls. Although this design improves clinical relevance for infertility-focused comparisons, minimal or asymptomatic endometriosis cannot be completely excluded among controls without surgical evaluation.

Third, serum TNF-α was measured at a single time point, and local inflammatory activity in peritoneal fluid or follicular fluid was not assessed. Fourth, previous surgical history and smoking status differed between groups. Although exploratory adjusted analyses were performed, the modest sample size limits model stability and residual confounding remains possible. Finally, no formal correction for multiple comparisons was applied; therefore, secondary and subgroup analyses should be interpreted as exploratory. Hormonal parameters were obtained on the third day of the menstrual cycle as part of routine infertility evaluation, which improved standardization for FSH, LH, and estradiol assessment.

Despite these limitations, the study has several strengths. It focused specifically on infertile women, used an infertile control group rather than healthy fertile controls, measured TNF-α using ELISA, and evaluated systemic inflammation together with AMH as a clinically relevant marker of ovarian reserve. The findings support the need for integrative approaches that combine inflammatory, hormonal, imaging, surgical, and reproductive outcome data rather than relying on a single systemic biomarker [15, 16].

## Conclusion

In this prospective comparative study, serum TNF-α levels were higher in infertile women with clinically and/or radiologically diagnosed endometriosis than in infertile controls, and this association persisted after adjustment for age, BMI, smoking status, and previous surgical history. However, serum TNF-α was not associated with infertility duration, AMH, or selected clinical characteristics. The unadjusted AMH difference was attenuated after adjustment, suggesting that ovarian reserve findings should be interpreted cautiously in the context of potential confounding, particularly previous surgery. Given the absence of surgical confirmation, lack of standardized staging, modest sample size, and absence of external validation, these findings should be considered exploratory. Serum TNF-α may reflect systemic inflammatory activity rather than infertility severity or ovarian reserve impairment.

## Data Availability

Availability of data and materials: The supplementary material supporting the conclusions of this article is available in the Zenodo repository, 10.5281/zenodo.19494117. The datasets generated and/or analyzed during the current study are available from the corresponding author on reasonable request.

## Abbreviations

TNF-α: Tumor necrosis factor-alpha
AMH: Anti-Müllerian hormone
FSH: Follicle-stimulating hormone
LH: Luteinizing hormone
E2: Estradiol
BMI: Body mass index
HSG: Hysterosalpingography
DIE: Deep infiltrating endometriosis
ELISA: Enzyme-linked immunosorbent assay
ROC: Receiver operating characteristic
AUC: Area under the curve
CI: Confidence interval

## References

[1] Becker CM, Bokor A, Heikinheimo O, Horne A, Jansen F, Kiesel L, et al. ESHRE guideline: endometriosis. Hum Reprod Open. 2022;2022(2):hoac009. 10.1093/hropen/hoac009. 35350465 10.1093/hropen/hoac009 PMC8951218

[2] Taylor HS, Kotlyar AM, Flores VA. Endometriosis is a chronic systemic disease: clinical challenges and novel innovations. Lancet. 2021;397(10276):839–52. 10.1016/S0140-6736(21)00389-5. 33640070 10.1016/S0140-6736(21)00389-5

[3] Horne AW, Missmer SA. Pathophysiology, diagnosis, and management of endometriosis. BMJ. 2022;379:e070750. 10.1136/bmj-2022-070750. 36375827 10.1136/bmj-2022-070750

[4] Bonavina G, Taylor HS. Endometriosis-associated infertility: From pathophysiology to tailored treatment. Front Endocrinol. 2022;13:1020827. 10.3389/fendo.2022.1020827. 10.3389/fendo.2022.1020827 PMC9643365 36387918

[5] Filip L, Duică F, Prădatu A, Crețoiu D, Suciu N, Crețoiu SM, et al. Endometriosis Associated Infertility: A Critical Review and Analysis on Etiopathogenesis and Therapeutic Approaches. Medicina. 2020;56(9):460. 10.3390/medicina56090460. 32916976 10.3390/medicina56090460 PMC7559069

[6] Miller JE, Ahn SH, Monsanto SP, Khalaj K, Koti M, Tayade C. Implications of immune dysfunction on endometriosis associated infertility. Oncotarget. 2017;8(4):7138–47. 10.18632/oncotarget.12577. 27740937 10.18632/oncotarget.12577 PMC5351695

[7] Galo S, Zúbor P, Szunyogh N, Kajo K, Macháleková K, Biringer K, et al. TNF-alpha serum levels in women with endometriosis: prospective clinical study. Ceska Gynekol. 2005;70(4):286–90. 16128129

[8] Tarokh M, Ghaffari Novin M, Poordast T, Tavana Z, Nazarian H, Norouzian M, et al. Serum and Peritoneal Fluid Cytokine Profiles in Infertile Women with Endometriosis. Iran J Immunol. 2019;16(2):151–62. 10.22034/IJI.2019.80258. 31182689 10.22034/IJI.2019.80258

[9] Falconer H, Sundqvist J, Gemzell-Danielsson K, von Schoultz B, D’Hooghe TM, Fried G. IVF outcome in women with endometriosis in relation to tumour necrosis factor and anti-Müllerian hormone. Reprod Biomed Online. 2009;18(4):582–8. 10.1016/s1472-6483(10)60138-1. 19401003 10.1016/s1472-6483(10)60138-1

[10] Anastasiu CV, Moga MA, Neculau AE, Bălan A, Scârneciu I, Dragomir RM, et al. Biomarkers for the Noninvasive Diagnosis of Endometriosis: State of the Art and Future Perspectives. Int J Mol Sci. 2020;21(5):1750. 10.3390/ijms21051750. 32143439 10.3390/ijms21051750 PMC7084761

[11] Krygere L, Jukna P, Jariene K, Drejeriene E. Diagnostic Potential of Cytokine Biomarkers in Endometriosis: Challenges and Insights. Biomedicines. 2024;12(12):2867. 10.3390/biomedicines12122867. 39767772 10.3390/biomedicines12122867 PMC11673701

[12] Othman EEDR, Hornung D, Salem HT, Khalifa EA, El-Metwally TH, Al-Hendy A. Serum cytokines as biomarkers for nonsurgical prediction of endometriosis. Eur J Obstet Gynecol Reprod Biol. 2008;137(2):240–6. 10.1016/j.ejogrb.2007.05.001. 17582674 10.1016/j.ejogrb.2007.05.001

[13] Kacem-Berjeb K, Braham M, Massoud CB, Hannachi H, Hamdoun M, Chtourou S, et al. Does Endometriosis Impact the Composition of Follicular Fluid in IL6 and AMH? A Case-Control Study. J Clin Med. 2023;12(5):1829. 10.3390/jcm12051829. 36902616 10.3390/jcm12051829 PMC10002901

[14] Han MT, Cheng W, Zhu R, Wu HH, Ding J, Zhao NN, et al. The cytokine profiles in follicular fluid and reproductive outcomes in women with endometriosis. Am J Reprod Immunol. 2023;89(6):e13633. 10.1111/aji.13633. 36250899 10.1111/aji.13633

[15] Kolanska K, Alijotas-Reig J, Cohen J, Cheloufi M, Selleret L, d’Argent E, et al. Endometriosis with infertility: A comprehensive review on the role of immune deregulation and immunomodulation therapy. Am J Reprod Immunol. 2021;85(3):e13384. 10.1111/aji.13384. 33278837 10.1111/aji.13384

[16] Fonseca BM, Pinto B, Costa L, Felgueira E, Rebelo I. Increased expression of NLRP3 inflammasome components in granulosa cells and follicular fluid interleukin(IL)-1beta and IL-18 levels in fresh IVF/ICSI cycles in women with endometriosis. J Assist Reprod Genet. 2023;40(1):191–9. 10.1007/s10815-022-02662-2. 36469254 10.1007/s10815-022-02662-2 PMC9840724

